# Comprehensive genomic analysis of *Xenorhabdus bovienii* strain MEL2.2

**DOI:** 10.1371/journal.pone.0331132

**Published:** 2025-08-25

**Authors:** Wipanee Meesil, Liam K. R. Sharkey, Sacha J. Pidot, Aunchalee Thanwisai

**Affiliations:** 1 Department of Microbiology and Parasitology, Faculty of Medical Science, Naresuan University, Phitsanulok, Thailand; 2 Department of Microbiology and Immunology, Doherty Institute, 792 Elizabeth Street, Melbourne, Australia; 3 Centre of Excellence in Medical Biotechnology (CEMB), Faculty of Medical Science, Naresuan University, Phitsanulok, Thailand; 4 Center of Excellence for Biodiversity, Faculty of Sciences, Naresuan University, Phitsanulok, Thailand; Chandigarh University, INDIA

## Abstract

The genome sequences of entomopathogenic bacteria and their functional analyses provide valuable insights for genetic engineering to enhance their use as biocontrol agents. In this study, we examine the draft genome of *Xenorhabdus bovienii* strain MEL2.2, which was isolated from entomopathogenic nematodes in Melbourne, Australia. The genome of *Xenorhabdus* strain MEL2.2 spans approximately 4.4 million base pairs and has a G + C content of 44.8%, aligning with known characteristics of the genus. Within the genome, 3,823 protein-coding genes were identified. Functional analysis revealed genes associated with nematode symbiosis and insect virulence. Moreover, 15 biosynthetic gene clusters (BGCs) were detected, potentially responsible for synthesizing various secondary metabolites. Comparative genomic analysis indicated a combination of conserved and strain-specific genes when compared to other *Xenorhabdus* *bovienii* strains, suggesting genetic traits that may enhance MEL2.2’s adaptability and pathogenicity. Altogether, these findings offer a foundation for exploring the strain’s utility in further applications.

## Introduction

The genera *Xenorhabdus* and *Photorhabdus* include entomopathogenic bacteria associated with *Steinernema* and *Heterorhabditis* nematodes, respectively [1,2]. These bacteria participate in mutualistic symbiotic relationships with nematodes, facilitating the infection and killing of insect hosts [3]. Beyond their ecological role, *Xenorhabdus* and *Photorhabdus* species are prolific producers of secondary metabolites with significant biotechnological and pharmaceutical potential. These bioactive compounds, including non-ribosomal peptides, polyketides, and other bioactive compounds, are synthesized by specialized biosynthetic gene clusters (BGCs) encoded within their genomes [4]. The metabolic versatility of these bacteria makes them valuable candidates for genome-guided studies [5,6] aimed at discovering novel bioactive compounds.

In Australia, various *Xenorhabdus* and *Photorhabdus* species have been isolated from several environments, emphasizing their ecological and biotechnological importance. Species include *X. miraniensis*, known for producing antibiotics such as xenocoumacin and xenorhabdin [7], *X. beddingii* [8], and *X. bovienii* strain T228, which is remarkable for its entomopathogenic properties and bioactive compound production, making it a strong candidate for biological pest control [8]. In addition, some *Photorhabdus* species, including

*P. luminescens* [9] and *P. asymbiotica*, have been implicated in human infections, particularly skin lesions [10]. Recently, the novel *P. viridis* was isolated from *Heterorhabditis zealandica* Poinar, 1990 [11] nematodes in Brecon, Australia [12], further highlighting the growing interest in these bacteria as promising sources of novel bioactive metabolites.

This study reports the draft genome of *X. bovienii* strain MEL2.2, with particular emphasis on genes related to symbiotic relationships, pathogenic mechanisms, and the biosynthesis of secondary metabolites. This genomic characterization expands our understanding of *Xenorhabdus* diversity and contributes to the ongoing exploration of microbial natural products.

## Methods

### Isolation of entomopathogenic bacteria

The strain MEL 2.2 was isolated from Melbourne, Australia. First, free-living infective juveniles (IJs) of entomopathogenic nematodes (EPNs) were isolated from soil samples using the baiting technique [13], in which *Galleria mellonella* (Linnaeus, 1758) [14] larvae were placed in a plastic box with soil samples and left for 5 days. Dead larvae were collected for EPN isolation. The White trap technique was then used to isolate EPNs from deceased *G. mellonella* larvae by placing them on filter paper in a petri dish setup with sterile distilled water, followed by incubation in the dark at room temperature for 15–20 days. However, no emerged EPNs were observed, possibly due to failed nematode reproduction within the cadavers. Potential contributing factors include insufficient nutrient availability, or suboptimal temperature conditions, any of which could hinder infective juvenile (IJ) development or emergence [15,16]. Subsequently, symbiotic bacteria associated with EPNs were then extracted from the hemolymph of *G. mellonella* larvae infected with IJs. The larvae were surface-sterilized with 70% ethanol, air-dried, and dissected using sterile forceps to expose the hemolymph, which was streaked onto nutrient bromothymol blue-triphenyltetrazolium chloride agar (NBTA). Plates were incubated in the dark at room temperature for 48–72 hours, after which a single colony was cultured in Luria-Bertani (LB) broth for 24 hours. The resulting cell pellet was then suspended in LB broth supplemented with 20% glycerol and stored at −80°C for further studies.

### Genome sequencing, assembly and annotation

A single colony was selected for DNA extraction using the GenFind V2 kit (Beckman Coulter). Illumina library preparation and short-read sequencing were performed at Doherty Applied Microbial Genomics, The University of Melbourne, Melbourne, Australia. Library preparation was conducted using the Illumina DNA Prep kit with quarter reagent volumes, followed by short-read sequencing on an Illumina NextSeq 500 with a 150 bp paired-end kit. The raw data were assessed for quality-checked using Falco (Version 1.2.4) [17]. Reads were trimmed with Trimmomatic (Version 0.39) [18]. Genome assembly was carried out with SPAdes (Version 3.15.5) [19], employing the --cov-cutoff parameter. Genome annotation was performed using Prokka (Version 1.14.6) [20]. Taxonomic classification was then performed using 16S rDNA and *recA* sequence analysis, along with Kraken2 [21]. Finally, QUAST [22] was used to assess data quality.

### Genomic analyses

Gene function was analyzed using the EggNOG 5.0-mapper tool [23], which mapped the sequences to the EggNOG database and assigned functional categories such as Clusters of Orthologous Genes (COGs). Briefly, FASTA gene sequences from Prokka were submitted via the EggNOG web interface. The results were then visualized using Python to display the functional distribution. Analysis of BGCs in strain MEL2.2 was conducted using AntiSMASH version 7.0 [24]. Following automated predictions, each detected SMBGC was manually curated. This involved rechecking the cluster boundaries, gene content, domain architecture, and alignment against the MIBiG database and previously characterized clusters to ensure functional accuracy. Pan and core genome analysis of available *Xenorhabdus* strains, retrieved from the NCBI database (S1 Table), was performed using Roary [25] and visualized using python.

## Results

### Isolation of entomopathogenic bacteria

**Fig 1:**
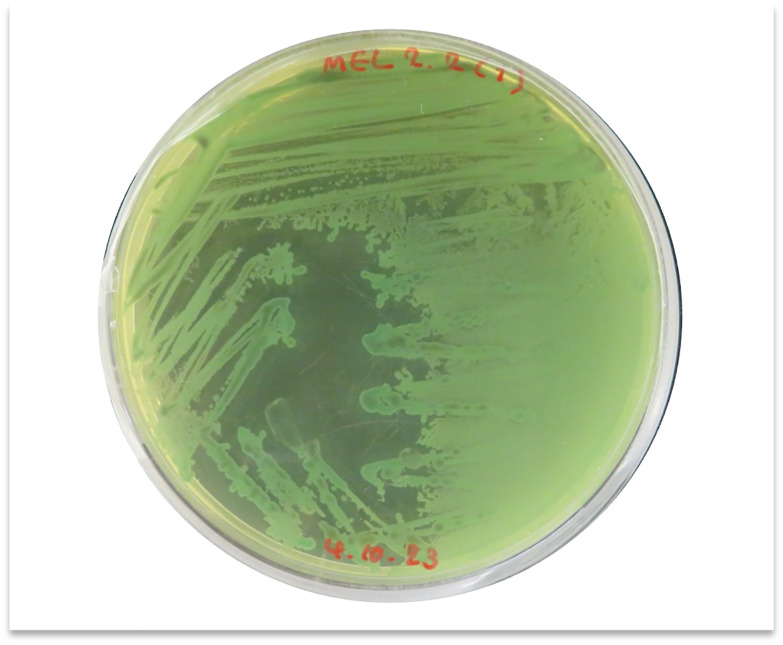
Colony characteristics of strain MEL 2.2 growing on NBTA.

During the baiting process, *G. mellonella* larvae exhibited mortality; however, no EPNs emerged from the dead larvae. Despite the absence of visible nematode emergence, a symbiotic bacterial strain, designated MEL2.2, was successfully isolated. After 72 hours of incubation, the strain developed distinct blue-green colonies on NBTA agar (Fig 1). Based on colony morphology and coloration, MEL 2.2 is likely a member of the genus *Xenorhabdus*, as *Xenorhabdus* species are known to produce blue colonies on NBTA agar [26].

### Genome sequencing, assembly and annotation

To characterize the genetic features of strain MEL2.2, its genome was sequenced using Illumina sequencing technology and submitted to NCBI (under the accession ID: SUB15210798). Assembly of the reads revealed the genome of strain MEL2.2 consisted of 284 contigs with an estimated total length of 4.4 Mbp, and an average G + C content of 44.8%. Annotation of the assembled genome predicted a total of 79 tRNA and 4 rRNA genes, with no plasmids detected. The genome comprises 3,823 coding sequences (CDS), of which 2,449 were functionally annotated, while 1,374 were classified as hypothetical proteins (Table 1). Taxonomic classification of strain MEL2.2 was performed using 16S sequence revealed 99% identity to *Xenorhabdus bovienii* T228.

**Table 1:** Genetic features of the *de novo* genome of the *Xenorhabdus bovienii* strain MEL 2.2.

Genome	*Xenorhabdus bovienii* strain MEL 2.2
Taxonomy	Bacteria; Pseudomonadota; Gammaproteobacteria; Enterobacterales; Morganellaceae; *Xenorhabdus*; *Xenorhabdus bovienii*; *Xenorhabdus bovienii* MEL 2.2
Size	4.4 Mb
GC Content	44.8%
Contigs	284
Contigs (>= 0 bp)	332
Contigs (>= 1000 bp)	248
Largest contig	1,24,360
N50	32784
N90	8846
L50	39
L90	133

**Fig 2:**
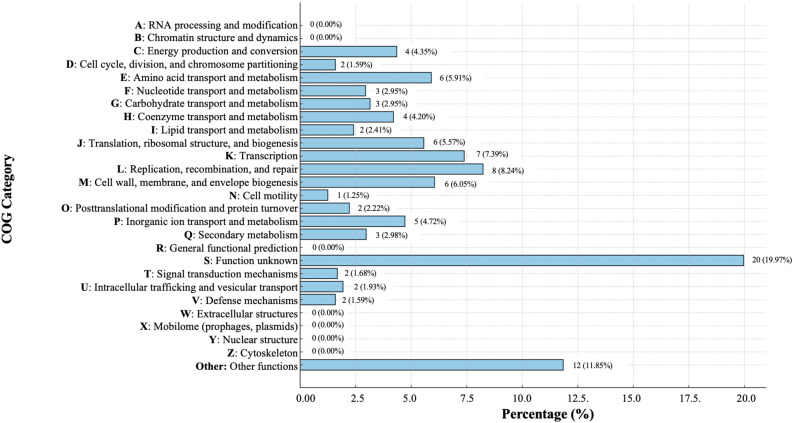
Distribution of gene features of *Xenorhabdus bovienii* strain MEL 2.2. The eggNOG functional classification statistical chart of functional genes shows the distribution of genes across different functional groups, represented from A to Z, along with their respective percentages.

Functional classification using eggNOG categories identified key gene categories (Fig 2 and S2 Table). Notably 290 proteins (8.24%) are involved in replication, recombination, and repair, while 260 proteins (7.39%) are associated with transcription. In addition, 213 proteins (6.05%) contribute to cell wall, membrane, and envelope biogenesis.

The genome also harbours 94 genes (2.67% of the total genome) related to virulence and symbiosis, which are primarily involved in toxin secretion, antibiotic resistance, and stress adaptation. Key virulence factors include resistance-nodulation-cell division (RND) efflux pumps (e.g., *mdtC*, *mdtB*), which contribute to multidrug resistance, and type I secretion system (T1SS) genes like *rtxD*, responsible for exporting toxins. Additionally, several toxin-antitoxin (TA) systems such as RelE, ParE, and MazF were identified, which play a role in bacterial persistence under stress conditions. Additionally, genes linked to insecticidal toxin production such as *tcaC*, a component of the toxin complex (Tc) [27], were identified, indicating potential involvement in insect pathogenesis.

### Antimicrobial and secondary metabolite potential

To investigate the biosynthetic gene clusters (BGCs) in *X. bovienii* strain MEL2.2, its genome was analyzed using AntiSMASH version 7.0 [24]. This analysis revealed 15 biosynthetic gene clusters (BGCs) spanning multiple secondary metabolite classes (Table 2). Comparative analysis using MIBiG and manual curation identified both known and novel biosynthetic pathways.

**Table 2:** The secondary metabolites of the *Xenorhabdus bovienii* strain MEL2.2.

Cluster	Type	Locus tag of core biosynthetics gene	Most similar known cluster	Identification Approaches	% Similarity	MIBiG accession/References
Cluster-1	NRPS	DMG_00274	Pyrrolizixenamide A	antiSMASH (MIBiG comparison)	43%	BGC0001873
Cluster-2	NRPS-like	DMG_00476	Unknown			
Cluster-3	NRPS	DMG_00674, DMG_00675	Taxlllaid A	antiSMASH (MIBiG comparison)	100%	BGC0001133
Cluster-4	Other	DMG_00717, DMG_00720	Unknown Thiopeptide			
Cluster-5	NRPS	DMG_00992	Xefoampeptides A-G	antiSMASH (Known Cluster Blast)	33%	BGC0001824
Cluster-6	NRPS	DMG_01272	Trichrysobactin	antiSMASH (Known Cluster Blast)	92%	BGC0002414
Cluster-7	NRPS	DMG_01535	Holomycin	antiSMASH (MIBiG comparison)	80%	BGC0002412
Cluster-8	NRPS	DMG_01707 - DMG_01709	Unknown			
Cluster-9	NRPS	DMG_01865, DMG_01866, DMG_01867	PAX	manual curation		[32,34]
Cluster-10	NRPS	DMG_02050	Bovienimide A	antiSMASH (MIBiG comparison)	100%	BGC0002135
Cluster-11	NRPS, T1PKS	DMG_02333 - DMG_02340	Unknown			
Cluster-12	PKS	DMG_02555, DMG_02556	Fabclavine-polyamine	antiSMASH (MIBiG comparison)	90%	BGC0001872
Cluster-13	Other	DMG_02814, DMG_02822	Putrebactin/avaroferrin	antiSMASH (Known Cluster Blast)	90%	BGC0001870
Cluster-14	Other (Betalactone)	DMG_03047, DMG_03049	Unknown			
Cluster-15	Other (PpyS-KS)	DMG_03163	Unknown			

Seven non-ribosomal peptide synthetase (NRPS) clusters were detected in the MEL 2.2 genome, exhibiting varying homology to known clusters (Table 2). These include clusters associated with the production of pyrrolizixenamide A [28], taxlllaid A [29], xefoampeptides A-G [4], trichrysobactin [30], holomycin [31], peptide-antimicrobial-*Xenorhabdus* (PAX) [32], and bovienimide A [33]. Among these, Cluster 9, predicted by antiSMASH as a NRPS, was identified as a putative peptide-antimicrobial-*Xenorhabdus* (PAX) cluster. This assignment was confirmed by manual curation. The predicted domain architecture of the NRPS enzymes within the cluster was re-examined and validated through comparison with previously characterized PAX clusters in *Xenorhabdus* spp. [32,34] which typically contains seven NRPS modules that together encode the biosynthesis of a heptapeptide. Each module includes canonical adenylation (A), thiolation (T), and condensation (C) domains, with some modules containing dual-function condensation/epimerization (C/E) domains. A terminal thioesterase (TE) domain catalyzes product release [32].

Additionally, the genome contains a polyketide cluster (Cluster 12) associated with fabclavine-polyamine (90% similarity) [35,36], and an NI-siderophore cluster (Cluster 13) linked to putrebactin/avaroferrin (90% similarity) [37]. In addition to these characterized clusters, six BGCs remain unclassified. Cluster 2 are small BGCs containing only three domains. Cluster 4 encodes an unknown thiopeptide with the *pflB* gene, which encodes pyruvate-formate lyase (PFL), a key enzyme linked to bacterial metabolism and virulence [38]. Cluster-8 and cluster-11 also lack similarity to any known BGC, suggesting novel biosynthetic potential. Meanwhile, Cluster 14 and Cluster 15 correspond to a betalactone-type cluster and a PpyS-KS cluster, respectively [24]. However, no characterized BGC with substantial homology was identified in MIBiG or KnownClusterBlast, and thus these clusters were classified as unknown.

### Comparative analysis

**Fig 3:**
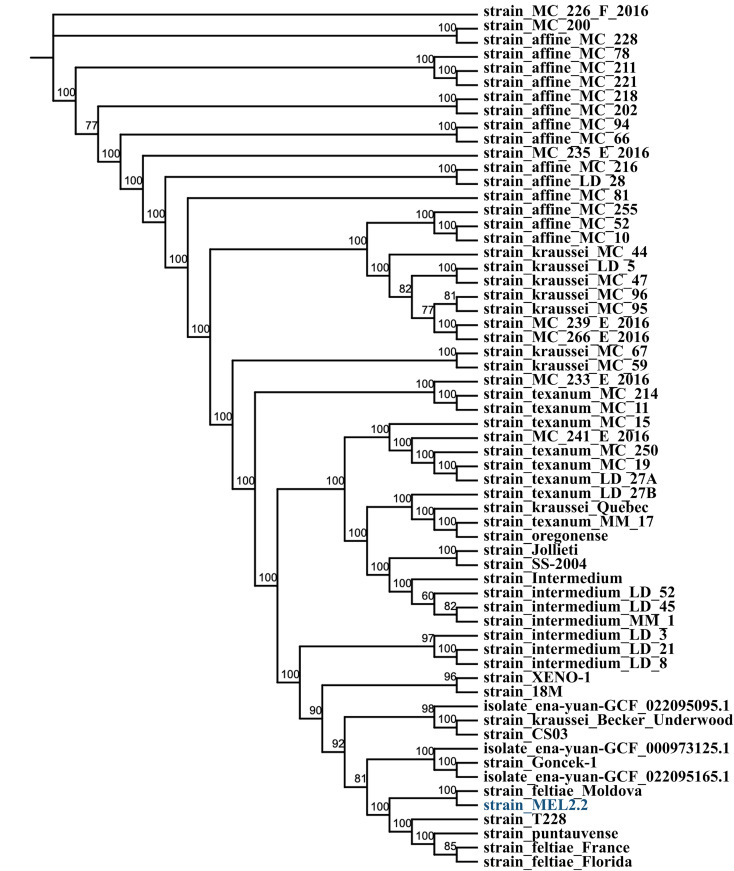
A phylogenetic tree of *Xenorhabdus bovienii* strains based on core gene alignment results from Roary program with 1,000 bootstraps.

**Fig 4:**
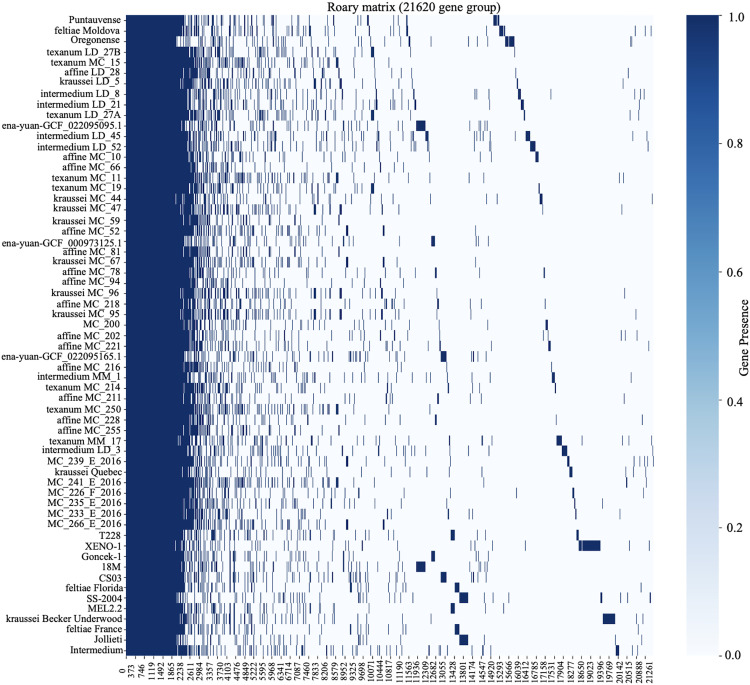
The pan and core genome heatmap of *Xenorhabdus bovienii* illustrates gene presence and absence across all strains. The x-axis represents orthologous groups, while the y-axis lists strains. The heatmap categorizes genes into core genes (highly conserved across strains), soft core genes (present in most but not all strains), shell genes (moderately conserved), and cloud genes (strain-specific or singletons). The distinct gene presence patterns suggest evolutionary relationships, genomic plasticity, and potential strain-specific adaptations, which may contribute to niche specialization, host interactions, or metabolic diversity.

To assess the genetic diversity among *X. bovienii* strains, a pan-genome analysis was conducted across 61 strains, including MEL2.2, using Roary [25]. The analysis identified four phylogenetic groups (Fig 3), revealing a total of 21,602 genes, highlighting the extensive genetic diversity within the species. Among these, 1,998 genes were classified as core genes, which are highly conserved across most strains, while 19,604 genes were categorized as accessory genes. These accessory genes were further divided into soft core, shell, and cloud genes, reflecting different levels of conservation (Fig 4) (Table 3).

**Table 3:** Summary of gene distribution across different categories based on their presence in all *Xenorhabdus bovienii* strains.

Gene Category	Description	Number of Genes
Core genes	(99% <= strains <= 100%)	1,998
Soft core genes	(95% <= strains < 99%)	224
Shell genes	(15% <= strains < 95%)	3,122
Cloud genes	(0% <= strains < 15%)	16,258
Total genes	(0% <= strains <= 100%)	21,602

Strain MEL2.2 shares a significant number of core genes with other *X. bovienii* strains but also possesses unique genes not found in several isolates (S3 Table). Notably, MEL2.2 exhibited considerable overlap with the *X. bovienii* strains SS-2004 and feltiae Floridam but lacked certain genes present in strains such as kraussei Becker Underwood and Jollieti. These genetic differences may contribute to niche-specific adaptations and functional variation within the species.

## Discussion

Successful isolation of strain MEL2.2 and subsequent identification revealed the strain to be a member of the species *X. bovienii*. Several *X. bovienii* strains have been isolated from various regions of the world, including Tasmania, Australia, the Czech Republic and Missouri, USA. While some of these isolates, such as SS-2004 from Missouri exhibited virulence against *Manduca sexta* Linnaeus, 1763 [39] and other insect species, others, such as CS03 from the Czech Republic were avirulent in insects [40,41]. Interestingly, *X. bovienii* strain MEL2.2 and these strains shared a high level of genetic similarity, suggesting there may be key genomic features influencing their biological functions, despite their isolation on different continents. Furthermore, their geographical origins also suggest potential adaptations to different environments and the broad dispersal and adaptation of *X. bovienii* worldwide. Further comparative genomic analysis is needed to determine the functional implications of these genetic relationships, particularly regarding insect pathogenicity and secondary metabolite production.

The key genome characteristics of *X. bovienii* strain MEL2.2 (genome size of approximately 4.4 Mbp, G + C content 44.8%), align well with the genomic characteristics of other *Xenorhabdus* species [40,42]. The genome encodes 3,823 coding sequences (CDS), of which 2,449 have functional annotations, while 1,374 (35.9%) are classified as hypothetical proteins. This large complement of hypothetical proteins suggests the presence of uncharacterized genes, which may contribute to unique metabolic pathways and ecological adaptation [43]. The identification of 79 tRNA and 4 rRNA genes highlights the strain’s robust translational capacity [44], while the absence of plasmids suggests that key virulence and metabolic traits are encoded within the chromosome [45].

Functional annotation indicated a notable enrichment in genes associated with replication, recombination, and repair processes (8.24%), suggesting a role in maintaining genomic integrity and facilitating adaptation under environmental stress conditions. [46,47]. Genes linked to transcription (7.39%) and to the biogenesis of the cell wall, membrane, and envelope (6.05%) highlight the strain’s capacity for dynamic gene regulation and preservation of structural integrity. Furthermore, 2.67% of the genome is associated with virulence and symbiosis, including genes encoding toxin-antitoxin systems, resistance-nodulation-cell division (RND) efflux pumps, and type I secretion systems (T1SS). These characteristics suggested that *X. bovienii* strain MEL2.2 possesses key traits that support persistence in various environments, potential antibiotic resistance, and the ability to interact with or manipulate its host [27,48].

The discovery of 15 biosynthetic gene clusters (BGCs) in *X. bovienii* strain MEL2.2 underscores its broad potential for secondary metabolites production. Among these, several known BGCs correspond to previously characterized compounds such as pyrrolizixenamide A, taxlllaid A, xefoampeptides A-G, PAX, and bovienimide A, which known for their antimicrobial effects, insecticidal properties, and contributions to symbiotic interactions [4,8]. Moreover, the presence of trichrysobactin suggested that MEL 2.2 harbors iron uptake, which are likely essential for its survival and virulence during insect infection. The ability to produce a wide range of antimicrobial compounds also enhance the bacterium’s effectiveness as a biocontrol agent by suppressing insect pathogens and microbial competitors. Additionally, the detection of the fabclavine-polyamine cluster, ts potent antimicrobial activity [35], further supports this potential as an effective biocontrol agent. The presence of putrebactin/avaroferrin BGC, previously reported in marine bacteria, implied that the strain possesses mechanisms for iron acquisition, which is vital for survival in iron-restricted environments like insect hemolymph [37,49].

Notably, the identification of six uncharacterized BGCs (Clusters 2, 4, 8, 11, 14, and 15) pointed to the potential for discovering novel secondary metabolites from strain MEL 2.2. Interestingly, cluster 4 encodes an unknown thiopeptide and includes the *pflB* gene, which is associated with pyruvate-formate lyase—an enzyme linked to bacterial metabolism and virulence [38]. This suggests a possible role in energy metabolism and stress adaptation, which may enhance bacterial survival under host-imposed stress conditions.

While antiSMASH provides valuable predictions of biosynthetic potential, experimental confirmation through metabolomics, gene expression analysis, or compound isolation remains essential to verify whether these BGCs are functionally active and under what conditions they are expressed. Previous studies have demonstrated that many BGCs, especially cryptic ones, require specific environmental cues or genetic activation to produce detectable metabolites [50–52]. Therefore, further research integrating metabolomic profiling, expression studies, and heterologous expression will be necessary to validate and characterize the natural products encoded by the BGCs identified in strain MEL2.2.

The pan and core genome analysis of *X. bovienii* highlights both conserved and unique genomic features. The presence of 1,998 core genes across 61 *X. bovienii* show the essential features that are required for members of this species, while three is significant variability in the accessory genome among different strains. This is most likely due to adaptation in diverse ecological niches among the different strains [53], and in particular, the metabolic pathways and hypothetical proteins present in MEL 2.2 suggest potential roles in specialized functions, possibly shaped by environmental pressures or host interactions. The similarity between *X. bovienii* strain MEL2.2 and *X. bovienii* strains such as SS-2004 and feltiae Florida points to shared ecological niches or evolutionary pressures despite geographic separation, indicating possible evolutionary relationships or functional convergence [53,54]. In contrast, variations observed when compared to strains such as kraussei Becker Underwood and Jollieti emphasize the genetic diversity present among different strains. This underscores the significant role of accessory genes in promoting strain-specific adaptation and diversity. Additional experimental studies focusing on the unique genes of MEL2.2 could provide valuable insights into its ecological functions and evolutionary background.

## Conclusion

In conclusion, genomic analysis of *X. bovienii* strain MEL2.2 revealed its genetic capacity for metabolism, virulence, and secondary metabolite production. Genes related to toxins, secretion systems, and antimicrobial biosynthesis support its dual as an insect pathogen and a nematode symbiont. Analysis of the pan and core genomes further emphasizes a conserved core genome essential for fundamental biological functions, alongside an accessory genome that provides strain-specific variability that contributes to adaptation to diverse ecological niches. These findings deepen our understanding of the strain’s ecological significance and underscore its promise for applications in biocontrol and biotechnology. Further investigation into the numerous hypothetical proteins and unexplored biosynthetic gene clusters could reveal novel characteristics and bioactive compounds with valuable practical uses.

### Declaration of Generative AI in Scientific Writing

The author used an AI-assisted tool (GPT-4 Omni) in some parts of the manuscript to correct English grammar.

## Supporting information

S1 Table *Xenorhabdus bovienii* genomes used in this study. (XLSX)

S2 Table Gene function of *Xenorhabdus bovienii* strain MEL 2.2 using the EggNOG 5.0-mapper tool. (XLSX)

S3 Table Pan and core genome analysis of 61 *Xenorhabdus bovienii* strains, including strain MEL2.2. (XLSX)
